# Topics of nuclear medicine research in Europe

**DOI:** 10.1007/s12149-017-1198-8

**Published:** 2017-07-25

**Authors:** Masayuki Inubushi, Tomohiro Kaneta, Takayoshi Ishimori, Etsuko Imabayashi, Atsutaka Okizaki, Naohiko Oku

**Affiliations:** 1 Division of Nuclear Medicine, Department of Radiology, Kawasaki Medical School, 577 Matsushima, Kurashiki, 701-0192 Japan; 2 Department of Radiology, Yokohama City University, 3-9 Fukuura, Kanazawa-ku, Yokohama, 236-0004 Japan; 3 Department of Diagnostic Imaging and Nuclear Medicine, Kyoto University Graduate School of Medicine, 54 Kawaharacho, Shogoin, Sakyoku, Kyoto, 606-8507 Japan; 4 Integrative Brain Imaging Center, National Center of Neurology and Psychiatry, 4-1-1 Ogawahigashi, Kodaira, 187-8551 Japan; 5 Department of Radiology, Asahikawa Medical University, 2-1-1-1 Midorigaoka-higashi, Asahikawa, 078-8510 Japan; 6 HIMEDIC Clinic WEST, 3-3-17 Minami Senba, Chuo-ku, Osaka, 542-0081 Japan

**Keywords:** Prostate-specific membrane antigen (PSMA), Radionuclide therapy, Dementia, Quantitative myocardial perfusion PET, Iodine-124 (^124^I)

## Abstract

Last year in the European Journal of Nuclear Medicine and Molecular Imaging, we introduced some recent nuclear medicine research conducted in Japan. This was favorably received by European readers in the main. This year we wish to focus on the Annals of Nuclear Medicine on some of the fine nuclear medicine research work executed in Europe recently. In the current review article, we take up five topics: prostate-specific membrane antigen imaging, recent advances in radionuclide therapy, [^18^F]fluorodeoxyglucose positron-emission tomography (PET) for dementia, quantitative PET assessment of myocardial perfusion, and iodine-124 (^124^I). Just at the most recent annual meeting of the European Association of Nuclear Medicine 2016, Kyoto was selected as the host city for the 2022 Congress of the World Federation of Nuclear Medicine and Biology. We hope that our continuous efforts to strengthen scientific cooperation between Europe and Japan will bring many European friends and a great success to the Kyoto meeting.

## Introduction

At the last annual meeting of the European Society of Nuclear Medicine 2016, prostate-specific membrane antigen (PSMA) imaging for prostate cancer (PCA) and somatostatin receptor imaging for neuroendocrine tumors (NET) attracted the most attention in their early clinical results and linkage to radionuclide therapy. Thus, we start the current review article by discussing recent oncological studies on PSMA imaging and radionuclide therapy.

Then, from two outstanding articles in neurology, we would like to reaffirm the fundamental and crucial roles of [^18^F]fluoro-deoxyglucose (FDG) positron-emission tomography (PET) not only for the clinical diagnosis and determination of prognosis of dementia, but also for measuring synaptic activity to investigate pathological changes.

As the next topic from cardiology, we take up quantitative PET assessment of myocardial perfusion. Although quantitative or semi-quantitative analysis is commonly used in neurological and oncological nuclear medicines, qualitative analysis has been a mainstay of diagnosis for ischemic heart disease or cardiomyopathy, because there are some difficulties specific in cardiac nuclear medicine imaging, such as relatively lower spatial resolution related to the size of the myocardium, and effects of artifacts derived from heartbeat, respiration, and physiological adjacent uptake. Nevertheless, the number of reports on quantitative PET assessment of myocardial perfusion has increased in recent years.

Finally, we focus on a relatively new radionuclide iodine-124 (^124^I). As a PET isotope, ^124^I is somewhat inferior to the traditional ones (e.g., ^18^F and ^11^C) in physical properties, but superior in biological and chemical characteristics. Giving a few recent instances of research application of ^124^I, we briefly discuss its potential future.

## Oncology—PSMA imaging

Prostate-specific membrane antigen, also known as folate hydrolase I or glutamate-carboxypeptidase II, is a type II transmembrane protein with glutamate-carboxypeptidase activity that shows a significant overexpression on PCA cells, but a low expression in normal tissues. Thus, PSMA can be considered as ideal as a target for radionuclide imaging and therapy for PCA. ^68^Ga-labeled PSMA-HBED-CC (PSMA-11) has been recently presented as a novel PET tracer for the detection of PCA recurrence and/or metastases. Unfortunately, PSMA-targeted PET imaging is not available in Japan yet, but this technique seems to have high clinical impact for the management of prostate cancer. Sterzing et al. [1] evaluated the potential of ^68^Ga-PSMA-11 PET/CT for the radiotherapeutic management, and reported that the TNM staging and therapy were changed in 50.8% of cases. Verburg et al. [2] reported that ^68^Ga-labeled PSMA-HBED-CC PET/CT identified PCA lesions even in patients with very low PSA levels. Positive findings were seen in 44, 79, and 89% of patients with PSA levels of ≤1, 1–2, and ≥2 ng/mL, respectively. Considering these results, PSMA-targeted PET imaging is not only specific for prostate cancer, but also extremely sensitive compared with the conventional imaging techniques, such as MRI, CT, bone scintigraphy, and FDG PET. The use of ^68^Ga generators and kits for radiosynthesis will make the tracer preparation easy and inexpensive. The cost-cutting of the tracer will promote the spread of ^68^Ga-PSMA-HBED-CC PET worldwide. PSMA-targeted radionuclide therapy using β- or α-emitters is also expected [3].

## Oncology—recent advances in radionuclide therapy

Radium-223 (^223^Ra) dichloride therapy is gaining widespread use in patients with symptomatic bone metastases from castration-resistant prostate cancer (mCRPC) in Europe and Japan. Although a survival benefit has been reported, it is not clear, where ^223^Ra should be placed in the treatment algorithm for mCRPC patients in the clinical setting. Etchebehere et al. [4] investigated the factors that may predict outcome in patients undergoing ^223^Ra therapy. In their study, the patients were able to tolerate chemotherapy and secondary hormonal therapy concomitant to ^223^Ra. Therefore, even in patients with visceral metastases, ^223^Ra can perhaps be considered alongside with chemotherapy, as there is a clear benefit in terms of overall survival (OS), progression free survival (PFS), and bone event-free survival (BeFS). In addition, abiraterone used concurrently with ^223^Ra seemed to have a positive effect in the patients, and the results of an ongoing randomized trial evaluating the use of abiraterone and ^223^Ra are expected. Since abiraterone is already approved in Japan, the results may alter the therapeutic strategy in the near future.

Peptide receptor radionuclide therapy (PRRT) for the treatment of NET has been studied for many years in Europe, although it is yet to be introduced in Japan. Mariniello et al. [5] investigated the long-term outcome of PRRT in 114 patients with advanced bronchopulmonary carcinoid (BPC). They compared the objective responses, OS, and PFS among three different PRRT protocols (^90^Y-DOTATOC vs. ^177^Lu-DOTATATE vs. ^90^Y-DOTATOC + ^177^Lu-DOTATATE). The median OS was 58.8 months and the median PFS was 28.0 months. ^177^Lu-DOTATATE protocol resulted in the highest 5-year OS (61.4%) and the ^90^Y-DOTATOC + ^177^Lu-DOTATATE protocol provided the highest response rate (38.1%). They concluded ^177^Lu-DOTATATE monotherapy to be the best option for PRRT, which proved to be promising in prolonging survival and delaying disease progression with the least toxicity. Since it is expected that PRRT using ^177^Lu-DOTATATE will be introduced in Japan in the near future, sophisticated prospective trials of Japanese patients will be required.

## Neurology—FDG PET for dementia

[^18^F]fluoro-deoxyglucose positron-emission tomography imaging has been used to investigate Alzheimer’s disease (AD), but few studies have attempted to discriminate between AD and non-AD dementia, instead focusing on patients with AD compared with control subjects. Perani et al. [6] have provided valuable insight into this issue. Cerebrospinal fluid (CSF) protein levels, structural magnetic resonance imaging (MRI), and FDG PET were used to examine patients with AD, frontotemporal lobar degeneration (FTLD), dementia with Lewy bodies (DLB), mild cognitive impairment (MCI) converters, and MCI nonconverters. In the differentiation of AD and non-AD, the CSF p-Tau/amyloid beta (Aβ) ratio showed 83% sensitivity and 64% specificity; FDG PET showed 94% sensitivity and 86% specificity. Inclusion of AD-related amyloid or tau pathology within the FTLD or DLB group reduced specificity compared with FDG PET. Furthermore, it is useful to discriminate FTLD or DLB, particularly with analysis using statistical parametrical mapping (SPM) t-maps. According to their statistical analysis of patients with MCI, FDG PET was the only predictor of conversion to AD in the final stepwise model.

Fluoro-deoxyglucose positron-emission tomography has shown that posterior cingulate cortex (PCC) hypometabolism is an indicator of the prodromal stages of AD [7]; however, the origin of this hypometabolism has not been elucidated completely. Teipel et al. [8] investigated this finding using cutting-edge image analysis. They measured the volume, amyloid load, and glucose metabolism in the hippocampus and PCC of subjects participating in the Alzheimer’s Disease Neuroimaging Initiative. They found that in cognitively normal individuals (CN) and those with early MCI (EMCI), PCC hypometabolism was associated only with hippocampus atrophy. In subjects with late MCI (LMCI), it was associated with both local and remote effects of atrophy as well as with local amyloid load. In subjects with AD dementia, PCC hypometabolism was related only to local atrophy. These findings suggest that the effects of remote pathology on PCC hypometabolism decrease and the effects of local pathology increase with progression from the preclinical to clinical stages of AD. Some functional MRI reports [9] described a link between amyloid pathology and disrupted network connectivity, suggesting that a decrease in the remote effect may have resulted from the disconnect, due to the local amyloid load in the PCC. Furthermore, in subjects with CN and EMCI, whose global amyloid load was low, they found a positive correlation between amyloid load and metabolism in the PCC. This result was consistent with one demonstrating that more synaptic activity led to higher levels of soluble Aβ species in ex vivo brain slices [10], because glucose metabolism is known to be a surrogate marker of synaptic activity.

## Cardiology—quantitative PET assessment of myocardial perfusion

Berti et al. [11] investigated the accuracy of segmental myocardial blood flow (MBF) and myocardial flow reserve (MFR) measurements assessed by quantitative cardiac PET imaging in the evaluation of coronary artery disease. The diagnostic accuracy of absolute segmental MBF/MFR values was assessed blinded to patients’ clinical data and to visual analysis of PET images, using certain MBF/MFR cut-off values. Inter-observer reproducibility of clinical decisions and the objective performance of MBF/MFR segmental values were also evaluated. 98 patients were included in the final analysis, and they underwent cardiac PET with H_2_^15^O, ^13^NH_3,_ or ^82^Rb. The gold standard was the findings of invasive and/or CT coronary angiography. The overall inter-observer agreements were 90% on a per-patient basis and 88% on a per-vessel basis. Segmental PET measurements correctly identified 85% of the patients. In vessel-based analyses, quantitative perfusion parameters had sensitivity, specificity, PPV and NPV of 92, 82, 42, and 99%, respectively. The authors concluded that the assessment of absolute myocardial perfusion parameters measured at a segment level leads to reliable and accurate identification of patients with significant coronary stenosis.

Castagnoli et al. [12] explored the role of quantitative myocardial PET for risk stratification in patients with hypertrophic cardiomyopathy (HCM). MBF <1.1 mL/min/g following dipyridamole (Dip-MBF) assessed by PET was previously identified as an important outcome predictor in HCM, although such extreme Dip-MBF impairment is only rarely observed recently. Therefore, the authors tried to reassess the Dip-MBF threshold for detecting high-risk HCM. Dip-MBF was measured using ^13^ N-ammonia in 100 patients with HCM. The endpoints were cardiovascular death and unfavorable outcome. The lowest tertile Dip-MBF was associated with a sevenfold independent risk of unfavorable outcome compared to the others. Dip-MBF 1.35 mL/min/g was identified as the best threshold for outcome prediction using a receiver-operating characteristics analysis. All cardiac deaths occurred in patients who had Dip-MBF impairment in the lateral wall. The authors concluded that Dip-MBF was a predictor of outcome in HCM, with the threshold for prediction higher than previously believed. Dip-MBF impairment in the lateral wall might be associated with cardiac death in HCM.

## Radionuclide—iodine-124

Iodine-124 (^124^I) is a positron-emitting nuclide of cyclotron product. As a PET isotope, ^124^I is somewhat inferior to the traditional ones (e.g., ^18^F and ^11^C) in physical properties. For ^124^I, positron decay ratio is low (only 23% of the time), positron energies are high (1352 and 2135 keV), and characteristic X-ray energies are also high (603 and 1691 keV). Low positron decay ratio results in the need for longer acquisition times as compared to the initial dose, and X-ray Compton scatters and wide positron range will lower image contrast.

However, iodine has biologically and chemically superior characteristics. First, iodine natively has high affinity with thyroid tissue. Binse et al. [13] clearly visualized metastatic lesions in patients with differentiated thyroid cancer (DTC) using ^124^I and PET/CT or PET/MRI. PET imaging using ^124^I will greatly contribute to the management of patients with high-risk DTC.

Second, radio iodination techniques are established in many substances including large molecules and proteins [14]. In the past decade, development of radio-labeled monoclonal antibodies (mAbs) has been accelerated by the need for targeted imaging and therapies. Especially, the relatively long half-life of ^124^I (4.2 days) tolerates a longer synthetic time and slower behavior of molecules in vivo. mAbs labeled with ^124^I will help visualize the biodistribution of the targeted antigen. Accurate quantitative information in high-resolution image can be obtained using PET. The analysis of the image can be readily applied to molecular diagnosis of diseases, dosimetry, and critical organ analysis. These basic simulative analyses are subsequently used in designing mAbs labeled with beta-emitting radionuclides such as ^131^I, ^186^Re, and ^90^Y.

These diagnostic and therapeutic techniques play important roles in the tailored treatment strategy for individual patients and in pharmaceutical development [15].

## Conclusion

Last year in the European Journal of Nuclear Medicine and Molecular Imaging (EJNMMI), we introduced some recent nuclear medicine research conducted in Japan [16], which was favorably received by the European readers in the main. This year we attempted to present in the Annals of Nuclear Medicine (ANM) some of the fine research work undertaken in Europe during the recent period. Just at the last annual meeting of the European Association of Nuclear Medicine (EANM) 2016, Kyoto was selected as the host city for the 2022 Congress of the World Federation of Nuclear Medicine and Biology (WFNMB). We hope that our continuous effort to strengthen scientific cooperation between Europe and Japan will bring many European friends and a great success to the Kyoto meeting.
